# Prevalence of burnout and contributing factors among physicians in Burundi: A cross-sectional study

**DOI:** 10.4102/sajpsychiatry.v32i0.2621

**Published:** 2026-02-24

**Authors:** Innocent Nduwimana, Epipode Ntawuyamara, Guoxia Zhang

**Affiliations:** 1 Department of Environmental Health, School of Public Health, Southern Medical University, Guangzhou, China; 2 Department of Environmental Health Promotion, Ministry of Public Health, Bujumbura, Burundi; 3 Department of Dermatology, Faculty of Cosmetology and Venereology, Shenzhen Hospital of Southern Medical University, Shenzhen, China; 4 Department of Dermatology and Venereology, Faculty of Medicine, Kamenge Teaching Hospital of the University of Burundi, Bujumbura, Burundi

**Keywords:** physician burnout, prevalence, contributing factors, Burundi, occupational stress, job satisfaction

## Abstract

**Background:**

Burnout negatively impacts interpersonal skills, job performance, career satisfaction and psychological health and is marked by physical and emotional exhaustion from prolonged exposure to emotionally demanding work. Despite its known consequences, research on physician burnout prevalence and burden in Burundi remains limited.

**Aim:**

This study aimed to assess the prevalence and investigate the contributing factors of burnout among physicians in Burundi.

**Setting:**

Public and private sector hospitals in Burundi.

**Methods:**

We conducted this cross-sectional analytical study in Burundi from 25 February 2025 to 25 April 2025, recruiting 435 hospital-based physicians with a minimum of 1 year of experience. Data on socio-demographics and burnout contributors were collected via a structured questionnaire, with burnout prevalence assessed using the Oldenburg Burnout Inventory (OLBI). SPSS was used for analysis. Multinomial logistic regression was used to investigate the association with burnout.

**Results:**

Among 435 physicians, burnout was high at 66.2%, with over half disengaged and most exhausted. Burnout was linked to heavy workloads, long hours, limited patient time, poor work-life balance, financial strain and difficult workplace dynamics, especially among younger, less experienced doctors.

**Conclusion:**

High prevalence rates of physician burnout have been observed in Burundi, driven by work-related and socio-demographic factors. Addressing the problem of burnout is crucial for sustainable healthcare, patient safety, physician well-being and overall healthcare system performance.

**Contribution:**

This study provides the first national-level evidence of physician burnout prevalence and contributing factors in Burundi, establishing a crucial foundation for targeted organisational interventions and developments.

## Introduction

Burnout syndrome has been a major concern widely discussed in the area of occupational health. It is described as a state of physical and emotional exhaustion resulting from extended exposure to a stressful and demanding work environment.^1^ This expansion of the scope of burnout has made it useful for describing the shared experience and stress of medical practice, particularly in conjunction with research demonstrating elevated levels of depressive symptoms among physicians.^2^ According to Maslach et al., there are three aspects of burnout: energetic (mental and physical tiredness), interpersonal (depersonalisation and cynicism) and assessment (negative appraisal of one’s own accomplishments).^3^

Professionals in high-stress occupations and those who frequently interact with the public, such as teachers, social workers and healthcare providers, are at increased risk of burnout.^4^ Physician burnout is widely recognised as a significant public health concern because of its adverse effects on patients’ care, doctors’ well-being and the healthcare system’s sustainability.^5,6^ There are many factors that contribute to the high levels of frustration among physicians worldwide. These include insufficient funding, excessive government and corporate oversight of healthcare delivery, sensationalised media portrayals of medical mistakes, unethical behaviour on the part of physicians, and challenges to their authority and expertise from both patients and other medical professionals.^7^

There are several challenges that physicians have on the job, including not getting enough sleep, dealing with patients’ demands for complete professionalism, balancing ethical principles with financial goals, and the constant threat of medical mistakes and malpractice lawsuits. Because of rising administrative burdens, less patient autonomy, and heavier caseloads, doctors’ work for patients has grown increasingly difficult in the last several decades.^8^ There are personal repercussions for doctors who experience burnout, but there are also potential negative effects on patient outcomes. Few studies have explored the effects of primary care physician burnout on patient satisfaction, yielding mixed results.^9^

Depending on the medical speciality and working conditions, the prevalence of burnout among healthcare workers internationally ranges from 15% to 85%.^10^ Numerous factors in healthcare organisations and systems contribute to this epidemic. These include heavy workloads, inefficient processes, clerical burdens, conflicts between work and home, physicians’ lack of control over issues affecting their work lives, organisational support systems and a leadership culture. Variables specific to each doctor also have an impact; for example, younger and female doctors tend to report greater rates of burnout.^11^ Recent national data suggest that 44% of United States (US) physicians experience symptoms of burnout, characterised by emotional tiredness and depersonalisation at least weekly. This finding confirms the well-documented incidence of physician discomfort recently.^12^

Medical professionals in Africa encounter numerous obstacles. The doctor-to-patient ratio is often inadequate, and there are many instances of overcrowding in emergency rooms, specialist clinics and outpatient departments.^13^ As a result of their doctors heavy workloads and long-standing gross neglect of their health, lower-middle-income countries urgently need this.^14^ It appears that burnout is becoming more common in Africa, with many of the same risk factors seen in the US, including an excessive workload, an imbalance between work and personal life, a lack of institutional support, and ineffective teamwork.^15^ The overall working population in high-income countries has a prevalence of burnout ranging from 13% to 27%.^16^ Furthermore, nearly 30% of locally qualified doctors in half of the sub-Saharan African nations have left for high-income countries.^17^ As a result, healthcare providers in sub-Saharan Africa are in short supply, and those who remain are more likely to burn out from caring for an abnormally high volume of critically ill patients.^16^ However, despite widespread concern regarding burnout, effectively mitigating its risk factors remains challenging because of a lack of detailed explanations of relevant contributing elements in current research.^18^

There is a significant dearth of data regarding the prevalence of physician burnout in Burundi. This knowledge gap hinders the development of targeted interventions and workplace policies aimed at mitigating burnout among healthcare professionals. Current research on this topic, particularly at the national level in Burundi, is scarce. This study directly addresses this deficit by assessing the prevalence and investigating the contributing factors of burnout among physicians in Burundi, thereby providing evidence to advocate for preventative measures.

## Research methods and design

### Study design, setting and participants

We conducted this analytical cross-sectional study (a design appropriate for estimating burnout prevalence and identifying associated factors at a single point in time) among physicians in Burundi. It is a Central African nation within the East African Community, from 25 February 2025 to 25 April 2025. Burundi’s healthcare infrastructure includes 1182 health centres – 658 public, 340 private, 144 confessional and 40 associations.^19^ The study included actively practising physicians from hospitals in Burundi with a minimum of 1 year of clinical experience. We only included physicians directly involved in patient care and able to provide informed consent.

### Sampling

The sample size was calculated using Cochran’s formula, which is suitable for large populations where precise demographic data are limited.

We calculated the sample size for this study using Cochran’s formula (Equation 1), a method justified by the limited availability of recent demographic data for Burundian physicians:


$$n=\frac{Z^{2}pq}{e^{2}};$$ [Eqn 1]

*e* = margin of error, equals 5%; *p* = proportion, equals 0.5; *q* = 1 – *p*; and *Z* = 1.96 at a confidence level of 95%.^20^

Then (Equation 2):


$$N_{0}=\frac{z^{2}pq}{e^{2}}=\frac{(1.96{)}^{2}(0.5)0.5}{(0.05{)}^{2}}=385$$ [Eqn 2]

To account for potential invalid or unreturned questionnaires, a 10% oversampling rate was applied.


$$\begin{array}{l} \text{There }\frac{10}{100}\times 385=38.5\;‡\;39,\\ \text{then }385+39=424(\text{Equation}3). \end{array}$$ [Eqn 3]

The study enrolled 435 physicians. Simple random sampling minimised selection bias and ensured broad physician representation; however, future studies could enhance precision by using stratified sampling across specialities and facility levels.

### Data collection

After obtaining informed consent, physicians were selected using simple random sampling and completed a structured questionnaire under the guidance of trained data collectors. Their presence ensured clarifications for unclear items and minimised incomplete responses.

The questionnaire captured socio-demographic characteristics, professional factors and known contributors to burnout identified in previous studies. Burnout was measured using the Oldenburg Burnout Inventory (OLBI),^21,22,23^ a validated 16-item tool assessing two theoretically grounded dimensions: exhaustion and disengagement. Each item was rated on a 5-point Likert scale (1 = strongly disagree to 5 = agree), with items 1, 5, 6, 8, 10, 13, 14 and 16 reverse-scored. To determine the overall level of burnout, the scores on the sub-scales (which ranged from 8 to 32) were added together. Higher scores mean that the person is more burnt out. Based on Leclercq et al., a total OLBI score of ≥ 44 indicates ‘burnout’ while scores < 44 indicate ‘no burnout’.^24^ While OLBI lacks universally accepted cutoffs, the chosen threshold is substantiated by empirical evidence and improves comparability with analogous studies.

The OLBI was chosen over the Maslach Burnout Inventory because of its broader conceptualisation of burnout, the use of both positive and negative item phrasing to reduce response bias, and the exclusion of dimensions (e.g., personal accomplishments) that were shown to behave inconsistently across populations.

### Validity and reliability of the questionnaire

To ensure data quality, the questionnaire underwent rigorous validation and reliability testing. An expert review led to improvements, followed by a questionnaire test with 10% of the sample to refine unclear questions. The pilot data yielded a Cronbach’s alpha of 0.742, indicating acceptable internal consistency. Validity was confirmed through content, construct and criterion measures, aligning with burnout literature and theoretical frameworks. Pilot testing also enhanced clarity and consistency, and test-retest methods confirmed response stability. This comprehensive approach strengthens the accuracy and meaningfulness of the data for robust findings on physician burnout in Burundi.

### Data analysis

Data analysis was conducted using Statistical Package for Social Sciences (SPSS) version 25. Descriptive statistics summarise categorical variables, while bivariate analyses generate crude odds ratios (CORs) with 95% confidence intervals (CIs) to assess unadjusted associations. Significant variables were entered into a multivariate model, where adjusted odds ratios (AORs) were estimated using multinomial logistic regression to identify independent predictors of burnout. Logistic regression was appropriate given the binary outcome and the need to control for confounders. Statistical significance was set at *p* < 0.05.

### Ethical considerations

An ethical approval certificate from the ethical committee of the University of Burundi was given prior to data collection under FM/CE04/M/2025 as a reference number. Furthermore, the clearance letter from the Office of the Ministry of Health was received, under reference number 633/121/DGSSLS/2025. All participants provided informed consent to participate after receiving comprehensive information regarding the study’s procedures. To safeguard participants’ anonymity, all direct identifiers (names and contact information) were pseudonymised using non-sequential codes, while quasi-identifiers, like specific hospital names and precise dates, were generalised into categories or redacted and replaced with generic descriptors in quotes to prevent reidentification. This study adhered to the ethical principles outlined in the Declaration of Helsinki regarding humans.

## Results

### Socio-demographic characteristics of study participants

Our study included 435 medical doctors, 375 (86.2%) male and 60 (13.8%) female among them.

As indicated in Table 1, the respondents in this study were primarily young, male and employed in public hospitals. Most participants were 30–40 years old (60.9%), male (86.2%) and married (66.7%). The majority worked in public hospitals (72.6%), with employment split between urban (48.0%) and rural (38.2%) settings. Common service units included internal medicine (19.8%) and in-patient consultation (19.5%). Furthermore, most participants had between 1 year and 10 years of professional experience (94.3%).

**TABLE 1:** Distribution of respondents by socio-demographic characteristics (*N* = 435).

Variables	Category	*n*	%
Age (years)	20–30	90	20.7
	30–40	265	60.9
	40–50	80	18.4
Work setting	Public hospital	316	72.6
	Private practice	85	19.5
	Confessional	34	7.8
Hospital location	Urban	209	48.0
	Semi-urban	60	13.8
	Rural	166	38.2
Gender	Male	375	86.2
	Female	60	13.8
Marital status	Married	290	66.7
	Single	140	32.2
	Divorced	5	1.1
Current working services	Emergency Department (ED)	79	18.2
	Internal medicine	86	19.8
	Paediatrics department	15	3.4
	Surgery department	76	17.5
	Obstetrics and Gynaecology Department	54	12.4
	Intensive Care Unit (ICU)	5	1.1
	In patient consultation	85	19.5
	Outpatient consultations	3	0.7
	Infectious and communicable disease department	15	3.4
	Psychiatry	5	1.1
	Medical imaging	5	1.1
	Ophthalmology	7	1.6
Years of experience	1–5	207	47.6
	5–10	203	46.7
	10–15	20	4.6
	More than 15	5	1.1

### Prevalence of burnout among respondents

We assessed the prevalence of burnout by considering the participants with a total score of equal to or higher than 44.

As presented in Table 2, the results indicate a high prevalence of burnout among the workforce, with 66.2% of respondents reporting experiencing burnout, while only 33.8% indicated its absence.

**TABLE 2:** Burnout status among physicians.

Score and interpretation	*n*	%
16–43: No burnout	147	33.8
44–64: Burnout	288	66.2

**Total**	**435**	**100.0**

In Table 3, the data reveal a significant prevalence of burnout within the surveyed workforce. Specifically, 53.3% of respondents reported experiencing disengagement, while a more substantial 71.3% reported experiencing exhaustion. Conversely, only 46.7% of respondents indicated an absence of disengagement, and 28.7% did not report feelings of exhaustion.

**TABLE 3:** Prevalence of burnout according to its sub-scales.

Score	Disengagement		Exhaustion	
	*n*	%	*n*	%
8–22	203	46.7	125	28.7
23–44	232	53.3	310	71.3

**Total**	**435**	**100.0**	**435**	**100.0**

### Factors contributing to physician burnout

Different factors contribute to burnout among our study population.

As indicated in Table 4, the data reveal significant challenges experienced by physicians. A substantial majority of respondents reported seeing over 25 patients daily (74.0%) and lacking sufficient time for patient care (88.3%). Workload is intense, with 38.6% working 60 h – 72 h per week and 83.7% experiencing a notable increase in workload over the past year. Patient consultation times are brief, with 83.0% spending less than 20 min per patient. Financial strain is pervasive, as 91.5% experience financial difficulties and 86.4% report associated anxiety. While most respondents reported positive relationships with colleagues and felt supported by friends and family, work-life balance remains a concern, with 32.4% struggling to manage competing promises.

**TABLE 4:** Distribution of respondents by factors contributing to physician burnout (*N* = 435).

Variables	Category	*n*	%
Number of patients seen in a workday	11–15	23	5.3
	16–20	53	12.2
	21–25	37	8.5
	More than 25	322	74.0
Working hours per week	Less than 60	126	29.0
	Between 60 and 72	168	38.6
	More than 72	141	32.4
Do you feel you have enough time to adequately care for each patient?	Yes	51	11.7
	No	384	88.7
Time of consultation for each patient (minutes)	More than 30	20	4.6
	Between 20 and 30	54	12.4
	Less than 20	361	83.0
How often are you required to work overtime or on-call shifts? (times per week)	1–2	182	41.8
	3–5	144	33.1
	More than 5	109	25.1
Compared to the past year, has your workload increased significantly?	Yes	364	83.7
	No	71	16.3
Do you have any flexibility in setting your work schedule?	Yes	224	51.5
	No	211	48.5
Number of shortages of necessary medications or equipment per month	1–3 times	219	50.3
	3–6 times	79	18.2
	More than 6 times	137	31.5
Relationships with colleagues	Well	144	33.1
	very well	270	62.1
	Not well	21	4.8
How well do you feel you can balance your work and personal life commitments?	Well	39	9.0
	Very well	232	53.3
	Not well	141	32.4
	Strongly not well	23	5.3
Do you feel you have a strong network of friends and family who can support you?	Yes	261	60.0
	No	174	40.0
Are you currently experiencing any financial difficulties?	Yes	398	91.5
	No	37	8.5
Level of satisfaction with the monthly income	Strongly unsatisfied	236	54.3
	Unsatisfied	158	36.3
	Neutral	34	7.8
	Satisfied	7	1.6
Does your financial situation cause you significant stress or anxiety?	Yes	376	86.4
	No	59	13.6

As shown in Table 5a and Table 5b, multivariable analysis indicated that several factors remained significant predictors of burnout. Seeing more than 25 patients per day nearly doubled burnout risk (AOR = 1.97, *p* < 0.001), while working fewer than 60 h per week was protective (AOR = 0.52, *p* = 0.001). A lack of schedule flexibility increased burnout odds twofold (AOR = 2.04, *p* = 0.027), and poor relationships with colleagues tripled the risk (AOR = 3.31, *p* < 0.001). Poor work-life balance was the strongest predictor, increasing burnout odds more than eightfold (AOR = 8.07, *p* < 0.001). Financial stress also showed a significant association (AOR = 0.23, *p* = 0.003). Together, these factors underscore workload, organisational conditions, interpersonal dynamics, work-life imbalance and financial pressure as key independent drivers of burnout.

**TABLE 5a:** Multinomial logistic regression of factors contributing to physician burnout in Burundi.

Variables	COR	95% CI	*p*	AOR	95% CI	*p*
Number of patients seen in a workday	1.460	1.178–1.808	**0.001**	1.973	1.386–2.809	**0.000**
Working hours per week	0.921	0.714–1.187	0.523	0.522	0.357–0.762	**0.001**
Do you feel you have enough time to adequately care for each patient?	3.581	1.960–6.544	**0.000**	1.463	0.527–4.060	0.465
Time of consultation for each patient (minutes)	1.049	0.714–1.542	0.807	0.934	0.446–1.956	0.857
How often are you required to work overtime or on-call shifts? (times per week)	1.604	1.236–2.082	**0.000**	1.267	0.821–1.955	0.284
Compared to the past year, has your workload increased significantly?	0.427	0.255–0.715	**0.001**	0.584	0.281–1.213	0.149
Do you have any flexibility in setting your work schedule?	2.459	1.627–3.717	**0.000**	2.036	1.084–3.823	**0.027**
Number of shortages of necessary medications or equipment per month	1.653	1.300–2.101	**0.000**	1.430	0.986–2.073	0.059
Relationships with colleague	5.222	3.436–7.936	**0.000**	3.309	1.851–5.916	**0.000**
How well do you feel you can balance your work and personal life commitments?	6.193	3.995–9.601	**0.000**	8.075	4.388–14.861	**0.000**
Do you feel you have a strong network of friends and family who can support you?	2.443	1.584–3.768	**0.000**	1.059	0.560–2.005	0.859
Are you currently experiencing any financial difficulties?	0.571	0.289–1.126	0.106	2.953	0.913–9.544	0.070
Level of satisfaction with the monthly income	0.385	0.283–0.522	**0.000**	0.660	0.418–1.043	0.075
Does your financial situation cause you significant stress or anxiety?	0.157	0.085–0.288	**0.000**	0.233	0.088–0.617	**0.003**
	-	-	-	-	-	-

Note: The bold values highlight findings that are statistically significant, providing reliable evidence of the relationships between the variables studied; The crude odds ratio is the unadjusted measure of how strongly one factor is linked to a yes/no outcome, based on a single-variable analysis; The adjusted odds ratio shows the relationship between a factor and an outcome after accounting for other influencing variables.

CI, confidence interval; COR, crude odds ratio; AOR, adjusted odds ratio.

**TABLE 5b:** Multinomial logistic regression of factors contributing to physician burnout in Burundi.

Variables	Category	No burnout		Burnout	
		*n*	%	*n*	%
Number of patients seen in a workday	11–15	13	56.5	10	43.5
	16–20	25	47.2	28	52.8
	21–25	14	37.8	23	62.2
	More than 25	95	29.5	227	70.5
Working hours per week	Less than 60	36	28.6	90	71.4
	Between 60 and 72	65	38.7	103	61.3
	More than 72	46	32.6	95	67.4
Do you feel you have enough time to adequately care for each patient?	Yes	31	60.8	20	39.2
	No	116	30.2	268	69.8
Time of consultation for each patient (minutes)	More than 30	4	20.0	16	80.0
	Between 20 and 30	25	46.3	29	53.7
	Less than 20	118	32.7	243	67.3
How often are you required to work overtime or on-call shifts? (times per week)	1–2	78	42.9	104	57.1
	3–5	44	30.6	100	69.4
	More than 5	25	22.9	84	77.1
Compared to the past year, has your workload increased significantly?	Yes	111	30.5	253	69.5
	No	36	50.7	35	49.3
Do you have any flexibility in setting your work schedule?	Yes	97	43.3	127	56.7
	No	50	23.7	161	76.3
Number of shortages of necessary medications or equipment per month	1–3 times	94	42.9	125	57.1
	3–6 times	23	29.1	56	70.9
	More than 6 times	30	21.9	107	78.1
Relationships with colleague	Well	85	59.0	59	41.0
	Very well	62	23.0	208	77.0
	Not well	0	0.0	21	100.0
How well do you feel you can balance your work and personal life commitments?	Well	29	74.4	10	25.6
	Very well	105	45.3	127	54.7
	Not well	13	9.2	128	90.8
	Strongly not well	0	0.0	23	100.0
Do you feel you have a strong network of friends and family who can support you?	Yes	108	41.4	153	58.6
	No	39	22.4	135	77.6
Are you currently experiencing any financial difficulties?	Yes	130	32.7	268	67.3
	No	17	45.9	20	54.1
Level of satisfaction with the monthly income	Strongly unsatisfied	56	23.7	180	76.3
	Unsatisfied	60	38.0	98	62.0
	Neutral	24	70.6	10	29.4
	Satisfied	7	100.0	0	0.0
Does your financial situation cause you significant stress or anxiety?	Yes	105	27.9	271	72.1
	No	42	71.2	17	28.8

As indicated in Table 6a and Table 6b, after adjustment, several socio-demographic factors remained significantly associated with burnout. Younger physicians (20–30 years) had more than twice the odds of burnout (AOR = 2.52, *p* = 0.001). Working in urban hospitals was protective compared with semi-urban and rural settings (AOR = 0.64, *p* = 0.001). Male physicians exhibited a greater risk of burnout compared to their female counterparts (AOR = 0.50, *p* = 0.021; reversed interpretation). Married physicians exhibited a heightened likelihood of experiencing burnout (AOR = 1.98, *p* = 0.044). Limited experience was a vulnerability factor, with physicians having only 1–5 years of practice showing higher burnout risk (AOR = 0.47, *p* = 0.002). These results highlight age, work location, gender, marital status and early-career stage as significant predictors of burnout.

**TABLE 6a:** Multinomial logistic regression on socio-demographic characteristics and burnout among Burundian physicians.

Variables	Burundi physician burnout					
	COR	95% CI	*p*	AOR	95% CI	*p*
Age (years)	1.256	0.912–1.730	0.163	2.522	1.432–4.439	**0.001**
Work setting	1.354	0.962–1.906	0.082	1.340	0.933–1.926	0.114
Hospital location	0.85	0.685–1.054	0.138	0.644	0.491–0.844	**0.001**
Gender	0.453	0.261–0.787	**0.005**	0.501	0.280–0.899	**0.021**
Marital status	1.119	0.748–1.612	0.585	1.977	1.018–3.841	**0.044**
Residence area	1.311	0.763–2.253	0.326	1.172	0.623–2.203	0.623
Current working services	0.984	0.915–1.059	0.675	0.993	0.918–1.075	0.867
Years of experience	0.781	0.573–1.065	0.118	0.473	0.296–0.756	**0.002**

Note: The bold values highlight findings that are statistically significant, providing reliable evidence of the relationships between the variables studied.

CI, confidence interval; COR, crude odds ratio; AOR, adjusted odds ratio.

**TABLE 6b:** Multinomial logistic regression on socio-demographic characteristics and burnout among Burundian physicians.

Variables	Category	Burundi physician burnout			
		No Burnout		Burnout	
		*n*	%	*n*	%
Age (years)	20–30	34	37.8	56	62.2
	30–40	91	34.3	174	65.7
	40–50	22	27.5	58	72.5
Work setting	Public hospital	115	36.4	201	63.6
	Private practice	23	27.1	62	72.9
	Confessional	9	26.5	25	73.5
Hospital location	Urban	67	32.1	142	67.9
	Semi-urban	14	23.3	46	76.7
	Rural	66	39.8	100	60.2
Gender	Male	117	31.2	258	68.8
	Female	30	50.0	30	50
Marital status	Married	99	34.1	191	65.9
	Single	48	34.3	92	65.7
	Divorced	0	0.0	5	100.0
Residence area	Urban	125	34.8	234	65.2
	Rural	22	28.9	54	71.1
Current working services	Emergency Department (ED)	18	22.8	127	56.7
	Internal medicine	41	47.7	161	76.3
	Paediatrics department	2	13.3	125	57.1
	Surgery department	36	47.4	56	70.9
	Obstetrics and Gynaecology department	8	14.8	107	78.1
	Intensive Care Unit (ICU)	0	0.0	59	41.0
	In patient consultation	30	35.2	208	77.0
	Outpatient consultation	0	0.0	21	100.0
	Infectious and communicable disease department	0	0.0	10	25.6
	Psychiatry	5	100.0	127	54.7
	Medical imaging	0	0.0	128	90.8
	Ophthalmology	7	100.0	23	100.0
Years of experience	1–5	64	30.9	143	69.1
	5–10	74	36.5	129	63.5
	10–15	4	20.0	16	80.0
	More than 15	5	100.0	0	0.0

## Discussion

Burnout constitutes a significant professional challenge, adversely affecting both individual performance and patient outcomes. In Burundi, physician burnout remains an understudied phenomenon, particularly at the national level. The Burundian physician workforce is characterised by a majority aged 30–40 years (60.9%), predominantly male (86.2%), and largely married (66.7%). The majority operate within public hospitals (72.6%), primarily in urban settings (48.0%). This demographic profile of a relatively young, male-dominated cohort heavily engaged in the public sector aligns with broader trends in healthcare workforces within many low- and middle-income countries, particularly in sub-Saharan Africa. In these regions, public health systems typically bear the primary responsibility for service delivery, and the representation of women in medicine may still be comparatively low. These findings are consistent with previous research indicating that younger physicians and those employed in public healthcare settings are more susceptible to higher rates of burnout, likely attributable to increased workload and occupational stress.^2^ Additionally, the relatively low proportion of physicians with over 15 years of experience (1.1%) suggests a need for further investigation into workforce retention strategies, as professional experience has been shown to influence burnout levels.^25^

The prevalence of burnout among physicians in Burundi is notably high, with 66.2% of respondents reportedly experiencing it. This figure aligns with global trends, indicating significant burnout rates among healthcare professionals worldwide.^26^ This level of burnout matches global estimates for public health workers, which usually fall between 10.5% and 66.2%,^26^ and is similar to a study in South Africa that found a burnout rate of 78%.^27^ A systematic review by Rotenstein et al. reported that overall burnout among physicians varies widely, ranging from 0% to 80.5%, highlighting the heterogeneity in burnout prevalence across different studies and regions.^2^ Another study by Karuna et al. found that burnout estimates ranged from 6% to 33%, further emphasising the variability in reported prevalence.^28^ Variations in reported burnout prevalence are often attributed to differences in the study populations. The high prevalence of burnout observed in Burundi is influenced by factors such as challenging working conditions, high patient loads and limited resources, all of which are known contributors to occupational burnout.^29^

This study on Burundian physicians reveals a significant prevalence of burnout symptoms: 53.3% reported disengagement, and 71.3% experienced exhaustion. The mean scores for disengagement (21.57) and exhaustion (23.64) indicate moderate to high levels of burnout, with a total mean score of 45.21 across both sub-scales. These results align with previous studies conducted in sub-Saharan Africa, where burnout rates among healthcare providers, particularly physicians, have been reported to be as high as 70%.^17^ For instance, a systematic review highlighted that burnout is prevalent (30% and 60%) among healthcare providers in various countries, with contributing factors including work environment, emotional distress and a lack of social support.^30^ Comparatively, the burnout rates observed in Burundi are consistent with findings from other regions, such as Portugal, where 66% of physicians reported high levels of emotional exhaustion.^5^ This suggests that the challenges confronting healthcare professionals in Burundi are reflective of broader systemic issues impacting physician well-being globally.

The OLBI is appropriate for this study because it measures exhaustion and disengagement – dimensions relevant to Burundian physicians’ working conditions. Its balanced item structure reduces response bias, and its cross-cultural suitability makes it effective in low-resource settings. The high scores observed confirm its sensitivity in detecting burnout; though its self-report nature may introduce subjectivity, suggesting the need for complementary methods in future research.

Analysis of burnout among Burundian physicians reveals that high patient load (70.5% burnout for those seeing > 25 patients/day), extended work hours (67.4% burnout for > 72 h/week), inadequate time for patient care (69.8% burnout) and poor work-life balance (90.8% burnout) are significantly associated with increased burnout levels. These findings align with previous studies that identify high patient volumes, extended working hours and poor work-life balance as prevalent contributors to burnout among healthcare providers in sub-Saharan Africa.^16^ This finding is in line with studies that emphasise the role of workplace flexibility in mitigating burnout.^27^ For instance, a systematic review highlighted that burnout rates among healthcare providers in this region can reach up to 70%, particularly among those contending with high workloads and inadequate support systems.^16^ Our findings from Burundi contribute to the growing body of literature on physician burnout in under-researched low-income countries, providing specific contextual data that largely corroborate the systemic drivers identified in broader global studies.

These findings highlight systemic pressures in Burundi’s health system, where staff shortages and limited resources increase workload. The strong link between poor work-life balance and burnout indicates a need for organisational measures – such as regulated hours or workload redistribution – to reduce stress among physicians.

Our study on physician burnout in Burundi shows significant associations with demographic and professional variables. Younger physicians (20–30 years old) exhibited a higher burnout rate (62.2%) compared to older colleagues, with an adjusted odds ratio (AOR = 2.52, *p* = 0.001). This aligns with previous studies indicating that younger healthcare professionals often experience greater stress because of inexperience and elevated expectations.^31^ While not statistically significant, burnout rates were notably high in private practices (72.9%) and confessional settings (73.5%). Conversely, urban hospital locations exhibited a higher prevalence of burnout (67.9%) compared to rural settings (60.2%). This finding suggests that the inherent pressures of urban healthcare environments may contribute to elevated stress levels among practitioners.^32^ We also observed gender differences, with male physicians exhibiting higher burnout prevalence (68.8%) compared to female physicians (50%). This finding is compatible with other African studies reporting higher burnout rates among male healthcare workers.^33^ Marital status further influenced burnout, with married physicians reporting a higher prevalence (65.9%) compared to single individuals (65.7%). This suggests that personal life dynamics may contribute to professional stress levels.^34^

Analysis stratified by years of experience revealed that physicians with 1–5 years of professional experience exhibited the highest burnout prevalence (69.1%). This observation may be attributable to the relatively young age of this cohort. The result may reflect the significant challenges faced by early-career physicians in adapting to the demands of the medical profession.^35^ It is possible these results are because of differences in the groups of people studied. More research is needed to better understand how factors such as age, gender and marital status affect how much doctors experience burnout.

### Scientific contribution and limitations

This study represents the first in-depth investigation into physicians’ burnout within Burundi, a significant strength given the limited existing research in the region. A key difference in this study is that it uses the OLBI instead of the more frequently used Maslach Burnout Inventory–Human Services Survey (MBI-HSS) found in similar research. Furthermore, the study quantifies the burden of burnout and identifies several contributing factors, providing valuable advice for developing preventative strategies aimed at mitigating it and enhancing physicians’ performance.

This research focuses on the high prevalence of burnout among physicians in Burundi, revealing a mix of work-related stressors and financial challenges as primary contributors. However, the findings are somewhat limited by the sample’s demographic makeup and the cross-sectional nature of the research. (The study predominantly sampled younger physicians, with very few physicians over the age of 50, which may underestimate the burnout risk among more experienced practitioners.) It was also limited to Burundi, meaning the results may not be generalisable to other African countries or regions with different healthcare infrastructures. Additionally, the study lacked any female representation, with only 13.8% of respondents being women, potentially skewing gender-specific burnout trends. The dependence on self-reported data, especially regarding burnout and financial stress, may lead to either underreporting or overreporting, contingent upon the physicians’ readiness to reveal their difficulties. Moreover, the cross-sectional design of the study limits its ability to determine causality between burnout and contributing factors, necessitating longitudinal studies for a more profound understanding of burnout’s long-term effects on physicians’ health and job performance. In summary, while the study highlights the high prevalence of burnout among physicians in Burundi, the findings are limited by the sample’s demographic makeup and the study’s design.

## Conclusion

This study aimed to determine the prevalence of physician burnout in Burundi and identify its contributing risk factors. We observed a high prevalence of burnout, primarily associated with work-related factors and specific socio-demographic characteristics.

These findings are crucial for informing public health policies. They underscore the necessity for measures to guide and monitor physicians, facilitating the early identification of those at risk for professional burnout. To enhance our understanding, future multicentric studies should incorporate a broader range of potential risk factors. In addition, researchers should consider implementing customised stress management programmes for physicians. Such initiatives could support early detection and timely intervention within the workplace, ultimately promoting physicians’ well-being.

## References

[1] Youssef D, Youssef J, Abou-Abbas L, Kawtharani M, Hassan H. Prevalence and correlates of burnout among physicians in a developing country facing multi-layered crises: A cross-sectional study. Sci Rep. 2022;12:12615. 10.1038/s41598-022-16095-5 35871153 PMC9308770

[2] Rotenstein LS, Torre M, Ramos MA, et al. Prevalence of burnout among physicians: A systematic review. J Am Med Assoc. 2018;320(11):1131–1150. 10.1001/jama.2018.12777 PMC6233645 30326495

[3] Bentulila Y, Lev Shalem L, Cohen B, Adler L. Physical work environment and burnout among primary care physicians in Israel: A cross-sectional study. BMC Prim Care. 2024;25:74. 10.1186/s12875-024-02310-x 38418978 PMC10900697

[4] Romani M, Ashkar K. Burnout among physicians. Libyan J Med. 2014;9:23556. 10.3402/ljm.v9.23556 24560380 PMC3929077

[5] Marques-Pinto A, Moreira S, Costa-Lopes R, Zózimo N, Vala J. Predictors of Burnout among physicians: Evidence from a national study in Portugal. Front Psychol. 2021;12:699974. 10.3389/fpsyg.2021.699974 34659015 PMC8517183

[6] Dinibutun SR. Factors affecting burnout and job satisfaction of physicians at public and private hospitals: A comparative analysis. J Healthc Leadersh. 2023;15:387–401. 10.2147/JHL.S440021 38077693 PMC10709107

[7] Kumar, S. Burnout and doctors: Prevalence, prevention and intervention. Healthcare (Basel). 2016;4(3):37. 10.3390/healthcare4030037 27417625 PMC5041038

[8] Wurm W, Vogel K, Holl A, et al. Depression-burnout overlap in physicians. PLoS One. 2016;11(3):e0149913. 10.1371/journal.pone.0149913 26930395 PMC4773131

[9] Chung S, Dillon EC, Meehan AE, Nordgren R, Frosch DL. The relationship between primary care physician burnout and patient-reported care experiences: A cross-sectional study. J Gen Intern Med. 2020;35:2357–2364. 10.1007/s11606-020-05770-w 32206992 PMC7403375

[10] Kim MH, Mazenga AC, Simon K, et al. Burnout and self-reported suboptimal patient care amongst health care workers providing HIV care in Malawi. PLoS One. 2018;13(2):e0192983. 10.1371/journal.pone.0192983 29466443 PMC5821338

[11] West CP, Dyrbye LN, Shanafelt TD. Physician burnout: Contributors, consequences and solutions. J Intern Med. 2018;283:516–529. 10.1111/joim.12752 29505159

[12] West CP, Dyrbye LN, Sinsky C, et al. Resilience and burnout among physicians and the general US working population. JAMA Netw Open. 2020;3:e209385. 10.1001/jamanetworkopen.2020.9385 32614425 PMC7333021

[13] Ayisi-Boateng NK, Bankah EM, Ofori-Amankwah GK, et al. A cross-sectional self-assessment of burnout amongst a sample of doctors in Ghana. Afr J Prim Health Care Fam Med. 2020;12:e1–e6. 10.4102/phcfm.v12i1.2336 PMC7479378 32896149

[14] Fernando BMS, Samaranayake DL. Burnout among postgraduate doctors in Colombo: Prevalence, associated factors and association with self-reported patient care. BMC Med Educ. 2019;19:373. 10.1186/s12909-019-1810-9 31619216 PMC6794729

[15] Iyer S, Suleman S, Qiu Y, Platt S. Risk factors for physician burnout: A perspective from Tanzania. Pan Afr Med J. 2020;41:298. 10.11604/pamj.2022.41.298.31055 PMC9250671 35855030

[16] Dubale BW, Friedman LE, Chemali Z, et al. Systematic review of burnout among healthcare providers in sub-Saharan Africa. BMC Public Health. 2019;19:1247. 10.1186/s12889-019-7566-7 31510975 PMC6737653

[17] Ahmed F, Hawulte B, Yuya M, Birhanu S, Oljira L. Prevalence of burnout and associated factors among health professionals working in public health facilities of Dire Dawa city administration, Eastern Ethiopia. Front Public Health. 2022;10:836654. 10.3389/fpubh.2022.836654 36033755 PMC9403244

[18] Edú-Valsania S, Laguía A, Moriano JA. Burnout: A review of theory and measurement. Int J Environ Res Public Health. 2022;19(3): 1780. 10.3390/ijerph19031780 35162802 PMC8834764

[19] Jean Marie N, Luximon-Ramma A, Jacques N. Analysis of health system for health security: Case of Burundi. Texila Int J Acad Res. 2024;11:16–28. 10.21522/TIJAR.2014.11.01.Art002

[20] Nanjundeswaraswamy TS, Divakar S. Determination of sample size and sampling methods in applied research. Proc Eng Sci. 2021;3:25–32. 10.24874/PES03.01.003

[21] Oana Tipa R, Tudose C, Lorin Pucarea V. Measuring burnout among psychiatric residents using the Oldenburg Burnout Inventory (OLBI) instrument. J Med Life. 2019;12:354–360. 10.25122/jml-2019-0089 32025253 PMC6993305

[22] Reis D, Xanthopoulou D, Tsaousis I. Measuring job and academic burnout with the Oldenburg Burnout Inventory (OLBI): Factorial invariance across samples and countries. Burn Res. 2015;2:8–18. 10.1016/j.burn.2014.11.001

[23] Kristensen TS, Borritz M, Villadsen E, Christensen KB. The Copenhagen burnout inventory: A new tool for the assessment of burnout. Work Stress. 2005;19:192–207. 10.1080/02678370500297720

[24] Leclercq C, Braeckman L, Firket P, Babic A, Hansez I. Interest of a joint use of two diagnostic tools of burnout: Comparison between the Oldenburg Burnout Inventory and the early detection tool of burnout completed by physicians. Int J Environ Res Public Health. 2021;18(19):10544. 10.3390/ijerph181910544 34639844 PMC8507986

[25] West CP, Dyrbye LN, Erwin PJ, Shanafelt TD. Interventions to prevent and reduce physician burnout: A systematic review and meta-analysis. Lancet. 2016;388:2272–2281. 10.1016/S0140-6736(16)31279-X 27692469

[26] Nagarajan R, Ramachandran P, Dilipkumar R, Kaur P. Global estimate of burnout among the public health workforce: A systematic review and meta-analysis. Hum Resour Health. 2024;22:30. 10.1186/s12960-024-00917-w 38773482 PMC11110232

[27] Khan S, Ntatamala I, Baatjies R, Adams, S. Prevalence and determinants of burnout among South African doctors during the COVID-19 pandemic. S Afr J Psychiatr. 2024;30:2225. 10.4102/sajpsychiatry.v30i0.2225 38726336 PMC11079362

[28] Karuna C, Palmer V, Scott A, Gunn J. Prevalence of burnout among GPs: A systematic review and meta-analysis. Br J Gen Pract. 2022;72:e316–e324. 10.3399/BJGP.2021.0441 34990391 PMC8869191

[29] Surawattanasakul V, Siviroj P, Kiratipaisarl W, et al. Physician burnout, associated factors, and their effects on work performance throughout first-year internships during the COVID-19 pandemic in Thailand: A cross-sectional study. BMC Public Health. 2025;25:1967. 10.1186/s12889-025-23172-7 40437382 PMC12117941

[30] Grotowska MŁ, Strawińska A, Wydro M. Burnout among physicians: Prevalence, contributing factors and solutions: A review of literature. J Pendidik Kedokter Indones: Indones J Med Educ. 2025;37:57716. 10.12775/QS.2024.37.57716

[31] Izdebski Z, Kozakiewicz A, Białorudzki M, Dec-Pietrowska J, Mazur J. Occupational burnout in healthcare workers, stress and other symptoms of work overload during the COVID-19 pandemic in Poland. Int J Environ Res Public Health. 2023;20(3): 2428. 10.3390/ijerph20032428 36767797 PMC9916221

[32] Batanda I. Prevalence of burnout among healthcare professionals: A survey at fort portal regional referral hospital. npj Ment Health Res. 2024;3:16. 10.1038/s44184-024-00061-2 38710834 PMC11074248

[33] Nwosu ADG, Ossai EN, Mba UC, et al. Physician burnout in Nigeria: A multicentre, cross-sectional study. BMC Health Serv Res. 2020;20:863. 10.1186/s12913-020-05710-8 32928201 PMC7489005

[34] Chen YH, Lou SZ, Yang CW, Tang HM, Lee CH, Jong GP. Effect of marriage on burnout among healthcare workers during the COVID-19 pandemic. Int J Environ Res Public Health. 2022;19(23):15811. 10.3390/ijerph192315811 36497885 PMC9737389

[35] Tanios M, Haberman D, Bouchard J, Motherwell M, Patel J. Analyses of burn-out among medical professionals and suggested solutions – A narrative review. J Hosp Manag Health Policy. 2021;6:7. 10.21037/jhmhp-20-153

